# Oral Administration of 5-Hydroxytryptophan Restores Gut Microbiota Dysbiosis in a Mouse Model of Depression

**DOI:** 10.3389/fmicb.2022.864571

**Published:** 2022-04-28

**Authors:** Lijuan Wu, Lisha Ran, Yazeng Wu, Manyu Liang, Jing Zeng, Famin Ke, Fang Wang, Jian Yang, Xiaoqing Lao, Li Liu, Qin Wang, Xiaowei Gao

**Affiliations:** ^1^Department of Endocrinology, The Affiliated Traditional Chinese Medicine Hospital of Southwest Medical University, Luzhou, China; ^2^School of Integrated Traditional Chinese and Western Medicine, Southwest Medical University, Luzhou, China; ^3^School of Pharmacy, Southwest Medical University, Luzhou, China; ^4^Institute of Microbiology, Jiangxi Academy of Sciences, Nanchang, China

**Keywords:** 5-Hydroxytryptophan, gut microbiome, depression, microbiota-gut-brain axis, short-chain fatty acids

## Abstract

5-Hydroxytryptophan (5-HTP) has positive clinical effects on various neuropsychiatric and metabiotic disorders, especially depression. Although it increases serotonin levels in the brain and gastrointestinal tract, its pharmacology remains largely unknown. Our goal was to determine the effects of 5-HTP on the mouse gut microbiome, which has a close relationship with depression through the “microbiota-gut-brain axis.” We confirmed that depressive disorder restructures the gut microbial community, and 5-HTP efficiently improves depressive symptoms in mice. Oral administration of 5-HTP significantly restored gut microbiota dysbiosis in mice with depression-like behaviors. The diversity and richness of gut microbial communities and relative abundance of specific microbial taxa at both phylum and genus levels were partially recovered. 5-HTP exhibited some positive effects on restoring the alterations in the concentrations of short-chain fatty acids and brain-derived neurotrophic factors caused by depression in mice. Our results may provide new insights into the pharmacology of 5-HTP in treating depression and other disorders.

## Introduction

Depression is the main neuropsychiatric disorder affecting more than 320 million people worldwide (World Health Organization, 2017). It strongly increases the risk of suicide and affects the quality of life of individuals. Moreover, depression has an important bidirectional relationship with other metabolic disorders, including obesity and diabetes (Chaves Filho et al., 2018). As predicted by the World Health Organization, depression is steadily advancing and will become a major public health and socioeconomic burden by 2030 (Mathers and Loncar, 2006). Although the exact etiology and pathophysiology of depression are still elusive, modern theories about the development of depression have involved monoaminergic system dysfunction, immune inflammation in the periphery and central nervous systems, and dysbiosis of the gastrointestinal microbiome (Capuco et al., 2020). Although some probiotics in the genera *Bifidobacterium* and *Lactobacillus* have demonstrated efficacy in improving depressive symptoms in both human and mouse models, the primary antidepressant drugs used in clinics are selective serotonin (5-HT) reuptake inhibitors (SSRIs), tricyclic antidepressants, norepinephrine reuptake inhibitors (SNRIs), and monoamine oxidase inhibitors, which can efficiently increase the monoamine levels in the synaptic cleft (Tian et al., 2019a; Meng et al., 2020).

In the human gut, there are a total of 10^14^-10^15^ bacterial cells distributed in 40,000 bacterial species, which form a complex ecosystem in terms of the “gut microbiome” and play important roles in food processing and digestion, immune system development, and maturation (Frank and Pace, 2008). After co-evolution with their host over millions of years, the gut microbiome establishes a close relationship with humans and substantially contributes to many aspects of physiological functions. As an extra “organ” in humans, dysbiosis of the gut microbiome can cause serious metabolic disorders and diseases, as revealed by numerous scientific and clinical investigations (Heintz-Buschart and Wilmes, 2018).

The term “gut-brain axis” describes the bidirectional communication between the gastrointestinal tract, enteric nervous system, and central nervous system (Mayer et al., 2015). Increasing evidence has demonstrated that the gut microbiome can modulate the gut-brain axis *via* neuroendocrine, neuroimmune, and neural pathways by directly or indirectly producing metabolites, including short-chain fatty acids (SCFAs) and tryptophan (Trp) metabolites (Chaves Filho et al., 2018). Remarkably, alterations in the gut microbiota composition in patients with depression have been observed, and transplanting the fecal microbiota from these patients to germ-free rats could induce depressive-like behaviors (Kelly et al., 2016). Disturbance in the equilibrium of gut microbiota contributes to the development of depression.

5-HTP, a precursor of 5-HT, can easily cross the blood-brain barrier without requiring a transporter and increase the brain 5-HT levels to yield an antidepressant-like effect (Birdsall, 1998). However, the rapid pharmacokinetics of 5-HTP with a half-life of ~2 h in humans make it impractical as a drug, and the neuropharmacology community turned their attention to the more effective and safe SSRIs (Turner et al., 2006). SSRIs can elevate the extracellular 5-HT levels in the brain by blocking the serotonin transporter (SERT) and thereby have an antidepressant function. According to the feedback from the clinic, the pharmacological potential of SSRIs is currently limited, and only a third of patients experience emotional improvement after treatment (Trivedi et al., 2006). Thus, inhibition of the activity of SERT by SSRIs alone might not be enough to elevate the extracellular 5-HT levels to elicit an antidepressant response in the brain, and treating depression with a second synergetic drug that elevates extracellular 5-HT levels beyond the effect of SSRIs could augment antidepressant efficacy Jacobsen J. P. et al. (2016) and Jacobsen et al. (2019). Recently, a slow-release formulation of 5-HTP with improved pharmacokinetic and pharmacodynamic properties was successfully developed and showed good therapeutic potential for depression when used with SSRIs (Jacobsen J. P. R. et al., 2016). This progress has made 5-HTP the focus of medical and scientific interest again.

In addition to treating depression, 5-HTP also shows positive clinical effects on various neuropsychiatric and metabolic disorders, including insomnia, chronic headaches, fibromyalgia, and obesity (Birdsall, 1998). Despite the capacity to increase the brain and gastrointestinal tract 5-HT levels after oral administration, the basic pharmacology of 5-HTP is still largely unknown. In this study, we investigated the effects of 5-HTP on the gut microbial composition of both healthy mice and mice with depression-like behaviors using 16S rDNA high-throughput sequencing. Our results confirmed significant disturbances in the gut microbiota composition of mice with depression-like behaviors. In healthy mice, the oral administration of 5-HTP only had limited effects on the gut microbiota community but substantially restored gut microbiota dysbiosis in mice with depression-like behaviors. The gut microbiome is correlated with several neuropsychiatric and metabiotic disorders, and the “microbiota-gut-brain axis” could modulate various central processes through the vagus nerve, microbial metabolites, and immune inflammation. Thus, our findings that 5-HTP unexpectedly exhibited the ability to regulate gut microbiota could provide important clues concerning the pharmacology of 5-HTP in the treatment of both neuropsychiatric and metabiotic disorders (Foster et al., 2017).

## Materials and Methods

### Mice and Reagents

Male C57BL/6J mice (5 weeks of age) were obtained from Chongqing Tengxin Biotechnology Co., Ltd. (Chongqing, China) and housed under standard conditions (12 h light-dark cycle, 24 ± 1°C, 55 ± 10% relative humidity) with free access to food and water. All experimental procedures involving animals were supervised and approved by the Southwest Medical University Ethics Committee (approval number: SWMU2021061). A normal commercially available diet (ND, 4.0% fat) and a high-fat diet (HFD, 35% fat) were purchased from Shanghai Huanyu Biotechnology Co., Ltd. (Shanghai, China) and used to feed the control and experimental mice, respectively. 5-HTP was purchased from Shanghai Aladdin Biochemical Technology Co., Ltd. (Shanghai, China). The enzyme-linked immunosorbent assay (ELISA) kits for mouse 5-HT (catalog no. ZC-37715W), brain-derived neurotrophic factor (BDNF) (catalog no. ZC-38512W), free fatty acid (FFA) (catalog no. ZC-38978W), and insulin (INS) (catalog no. ZC-38920W) were obtained from Shanghai ZCIBIO Technology Co., Ltd. (Shanghai, China). The standards of SCFAs used for GC-MS analysis were purchased from Dr. Ehrenstorfer GmbH (Augsburg, Germany).

### Experimental Design

**Figure 1:**
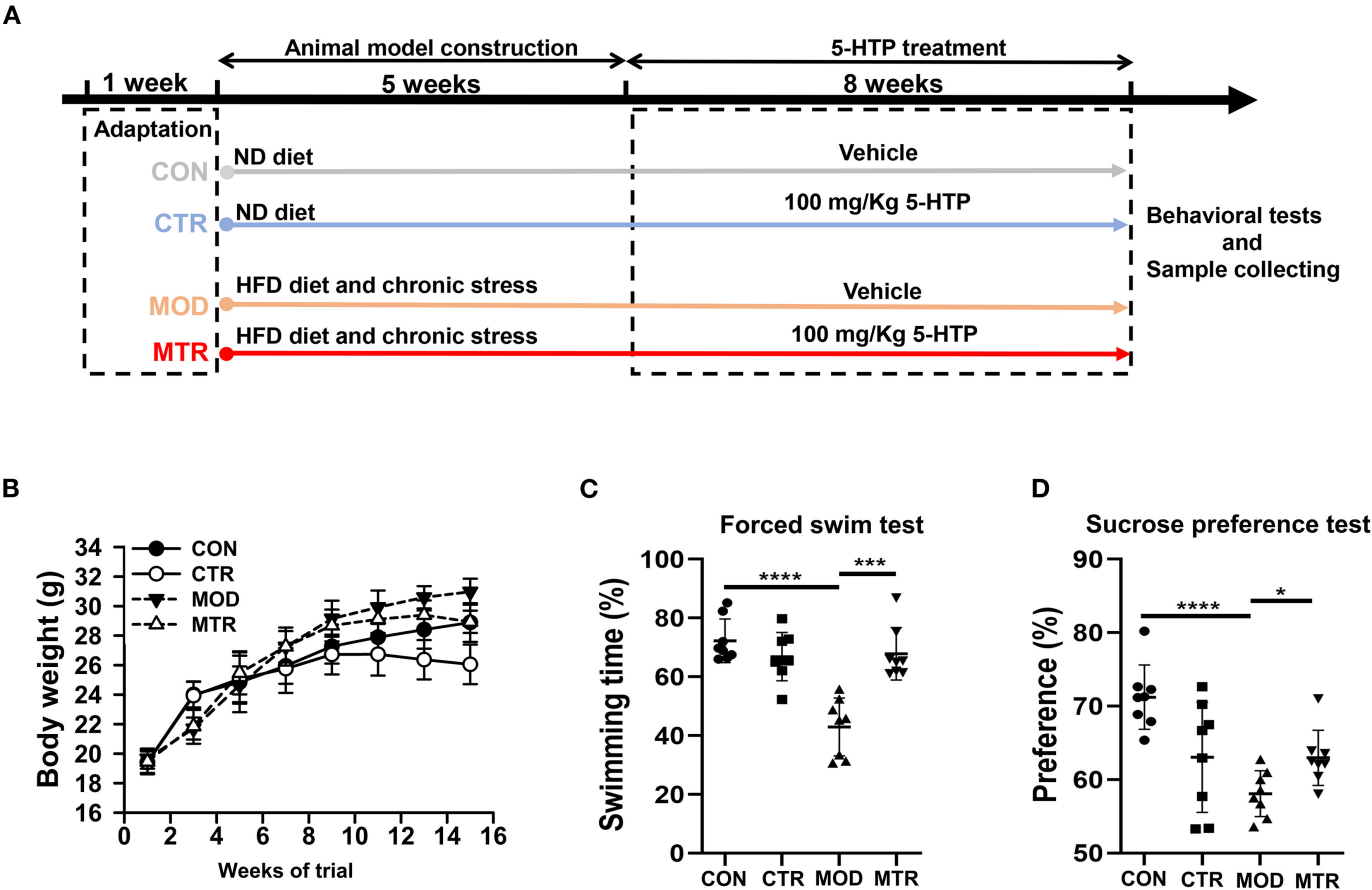
Animal experiment schedule and behavioral tests in the four groups. **(A)** Animal experiment schedule. The experimental period lasted for 14 weeks, including 1 week for adaptation, 5 weeks for the induction of depressive disorders, and 8 weeks for 5-HTP treatment. **(B)** Body weight of the mice in each group. Mice were weighed every 2 weeks after beginning the experiment. **(C)** Forced swim test. The mice were placed in a glass tank filled with 20 cm deep water at 25 ± 1°C. The continuous swimming time of the mice was recorded using a video tracking system. The percentage of swimming time in the 4 min experiment was calculated. **(D)** Sucrose preference test. Before testing, the mice were habituated to sucrose and water bottles randomly for 72 h. After acclimatization, mice were water fasted for 8 h, then separated into individual cages equipped with two drinking bottles for 2 h: one containing 1% sucrose and the other containing water. Bottles were weighed before and after the test. Sucrose preference = [Sucrose solution intake/(Sucrose solution + Water) intake] ×100%. Data are expressed as the mean with SD. *p* < 0.05 was considered statistically significant (*p < 0.05, ***p < 0.005, and ****p < 0.0001).

In this study, we used both the HFD diet and chronic unpredictable stress to induce the depression phenotype in mice (Vagena et al., 2019; Tian et al., 2020). The experimental schedule used in this study is shown in Figure 1A. Forty C57BL/6J mice with similar weights were randomly and equally allocated into four groups: the control group (CON), control group treated with 5-HTP (CTR), depression model group (MOD), and depression model group treated with 5-HTP (MTR). The dose of 5-HTP used in this study was 100 mg kg^−1^ per day as reported by Jacobsen J. P. et al. (2016) and Jacobsen et al. (2019). Before conducting the experiments, all mice were fed an ND diet for 1 week while adapting to their environment. The CON and CTR groups were fed the ND diet during the experiment. At the 7th week, the CON group mice were orally gavaged with distilled water, whereas the CTR group was orally gavaged daily with 5-HTP in distilled water for another 8 weeks. The MOD and MTR groups were fed the HFD diet throughout the experiment. At the 2nd week, both groups began to receive two chronic and unpredictable mild stressors daily for 4 weeks, followed by a chronic and unpredictable mild stressor daily for another week. The detailed protocols for chronic and unpredictable mild stressors could be found in the Supplementary Material. At the 7th week, the MOD group was orally gavaged with distilled water, whereas the MTR group was orally gavaged daily with 5-HTP in distilled water for another 8 weeks. At the end of the experiment, feces, blood serum, and hippocampus samples of mice were collected in sterilized tubes and stored at −80 °C for further analysis.

### Bacterial DNA Extraction and 16S rRNA Gene Sequencing

Bacterial DNA was extracted from the mouse fecal samples using the E.Z.N.A. Stool DNA Kit (OMEGA, Bio-Tek, USA) according to the manufacturer's instructions. The quality of the resulting DNA was analyzed by 1% agarose gel electrophoresis, and the concentrations were determined using a NanoDrop instrument (Thermo Fisher Scientific, Wilmington, DE, USA). Using the extracted bacterial genomic DNA as a template, the hypervariable regions (V3/V4) of the 16S rRNA gene were amplified using the 338F/806R primer pair. The sequences of the primer pairs were as follows: 338F (5′-ACTCCTACGGGAGGCAGCAG-3′) and 806R (5′ -GGACTACHVGGGTWTCTAAT-3′). After amplification, the PCR samples were subjected to 2% agarose gel electrophoresis, and the DNA products were purified using a DNA Gel Extraction Kit (OMEGA, Bio-tek, United States). After quantification, the purified amplicons were mixed in equimolar ratios and paired-end sequenced on an Illumina MiSeq platform (Illumina, San Diego, USA) at Majorbio Bio-Pharm Technology Co., Ltd. (Shanghai, China).

### Sequence Processing and Bioinformatic Analysis

The raw sequences obtained in this study were submitted to the National Center for Biotechnology Information short-read archive database (accession number: PRJNA782400). Bioinformatic analysis of the gut microbiota community was conducted using the free online i-Sanger Cloud Platform of Majorbio (www.i-sanger.com). The raw reads were demultiplexed, quality-filtered using Trimmomatic software, and merged using FLASH 2 with the criteria described previously (Wu et al., 2020). To clarify the gut microbiota composition and diversity, operational taxonomic unit (OTU) analysis was performed using the Quantitative Insights Into Microbial Ecology (QIIME) toolkit (version 1.17), and OTUs were clustered with a threshold of 97% similarity using UPARSE (version 7.1). The representative OTUs were annotated with taxonomic information using the RDP Classifier algorithm (http://rdp.cme.msu.edu/) against the SILVA ribosomal RNA gene database with a confidence threshold of 70%.

Rarefaction curves were generated by plotting the number of OTUs against the number of identified sequences and were used to estimate whether the sequences obtained from each sample were sufficient for species identification. The alpha- and beta-diversity analyses of the gut microbiota community in each sample were conducted using Mothur (version 1.30.1) and QIIME 2 software, respectively. Hierarchical clustering tree, weighted UniFrac-based principal coordinate analysis (PCoA), and partial least squares discriminant analysis (PLS-DA) were conducted using the R software package. Linear discriminant analysis (LDA) coupled with effect size (LEfSe) was performed using the LEfSe program. Non-parametric one-way analysis and Wilcoxon rank-sum test were used to determine the statistical significance of differences in microbial composition at both the phylum and genus levels among the four groups. The main differences at the genus level in each sample were revealed using hierarchical clustering heatmap analysis. Functional changes in the gut microbiota of the four groups were predicted by mapping the 16S rRNA sequencing data against the Kyoto Encyclopedia of Genes and Genomes (KEGG) database using the PICROSt software package.

### Determination of SCFAs

The analysis of SCFAs (acetic, propionic, isobutyric, butyric, isovaleric, valeric, hexanoic, heptanoic, octanoic, nonanoic, and decanoic acid) was performed using a Shimadzu GC2030-QP2020 NX gas chromatography-mass spectrometer (GC-MS) equipped with an HP-FFAP capillary column. Serum samples (100 μL), 50 μL 50% H_2_SO_4_, and 200 μL 25 mg L^−1^ 2-methyl valeric acid (internal standard) were mixed into sterile tubes, followed by sonication on ice for 10 min. The resulting supernatants were used for GC-MS analysis after centrifugation at 10,000 × *g* and 4°C for 15 min and maintenance at −20°C for 30 min. A 1 μL aliquot of the analyte was injected in split mode (5:1). Helium was used as the carrier gas, the front inlet purge flow was 3 mL min^−1^, and the gas flow rate through the column was 1 mL min^−1^. The initial temperature was maintained at 80°C for 1 min, then raised to 200°C at a rate of 10°C min^−1^ for 5 min, and then increased for 1 min to 240°C at a rate of 40°C min^−1^. The injection, transfer line, quad, and ion source temperatures were 240, 240, 200, and 150°C, respectively. The energy was −70 eV in the electron impact mode. The mass spectrometry data were acquired in Scan/SIM mode with an m/z range of 33–150 after a solvent delay of 3.5 min.

### ELISA Analysis

The concentrations of 5-HT and BDNF in the hippocampus and FFA and insulin in serum were measured using ELISA kits according to the manufacturer's instructions (ZCIBIO, China). Briefly, the samples were incubated in 96-well plates with the corresponding anti-mouse antibodies at 37°C for 1 h. After washing three times with buffer, horseradish peroxidase-conjugated antibodies for the different samples were added to the wells to bind the targets. Then, the amount of the targets in each well was reflected by the visible color converted from the colorimetric substrate catalyzed by horseradish peroxidase. The colorimetric signals were determined using a plate reader at OD_450_ nm, and the final concentration was calculated based on a standard curve.

### Statistical Analysis

Unless otherwise indicated, GraphPad Prism software (version 8.0; USA) was used for statistical analysis. A one-way ANOVA with Dunnett's test or *post-hoc* Tukey-Kramer test were applied for comparisons. All data were considered statistically significant at *p* < 0.05.

## Results

### Effects of 5-HTP on the Body Weight and Behavior of Mice With Depression-Like Behaviors

Figure 1B shows the effects of oral administration of 5-HTP on the body weights of both healthy mice and mice with depression-like behaviors. The MOD group had a higher body weight than the CON group, and the MTR group had a higher body weight than the CTR group at the end of the experiment. This might have occurred because heavier mice were fed the HFD diet. The body weights of the two 5-HTP treated groups were lower than those of the corresponding 5-HTP untreated groups: the CTR and the CON group, and the MTR and the MOD group, indicating that oral administration of 5-HTP could reduce the body weights of both healthy mice and mice with depression-like behaviors. According to the behavioral test results, the MOD group showed significantly decreased swimming time and sucrose preference than the CON group (Figures 1C,D). Compared with the MOD group, the mice in the MTR group had improved swimming time and sucrose preference, which may have occurred because of the positive effects of oral administration of 5-HTP on the depressive disorder in mice.

### Effects of 5-HTP on the Gut Microbiota of Mice With Depression-Like Behaviors

#### Characteristics of the High-Throughput Sequencing Data

After filtering low-quality reads according to the criteria described previously, a total of 1,485,113 valid sequences with an average length of 419 were obtained. Detailed information on the valid reads from 32 samples is shown in Supplementary Table 1. The Good's coverage index was ≥99%, and the rarefaction curve reached a saturation plateau, which indicated that the sequencing depth was sufficient to identify most of the bacterial phylotypes in each sample (Supplementary Table 1; Supplementary Figure 1).

#### Diversity Analysis of the Gut Microbiota in Different Groups

**Figure 2:**
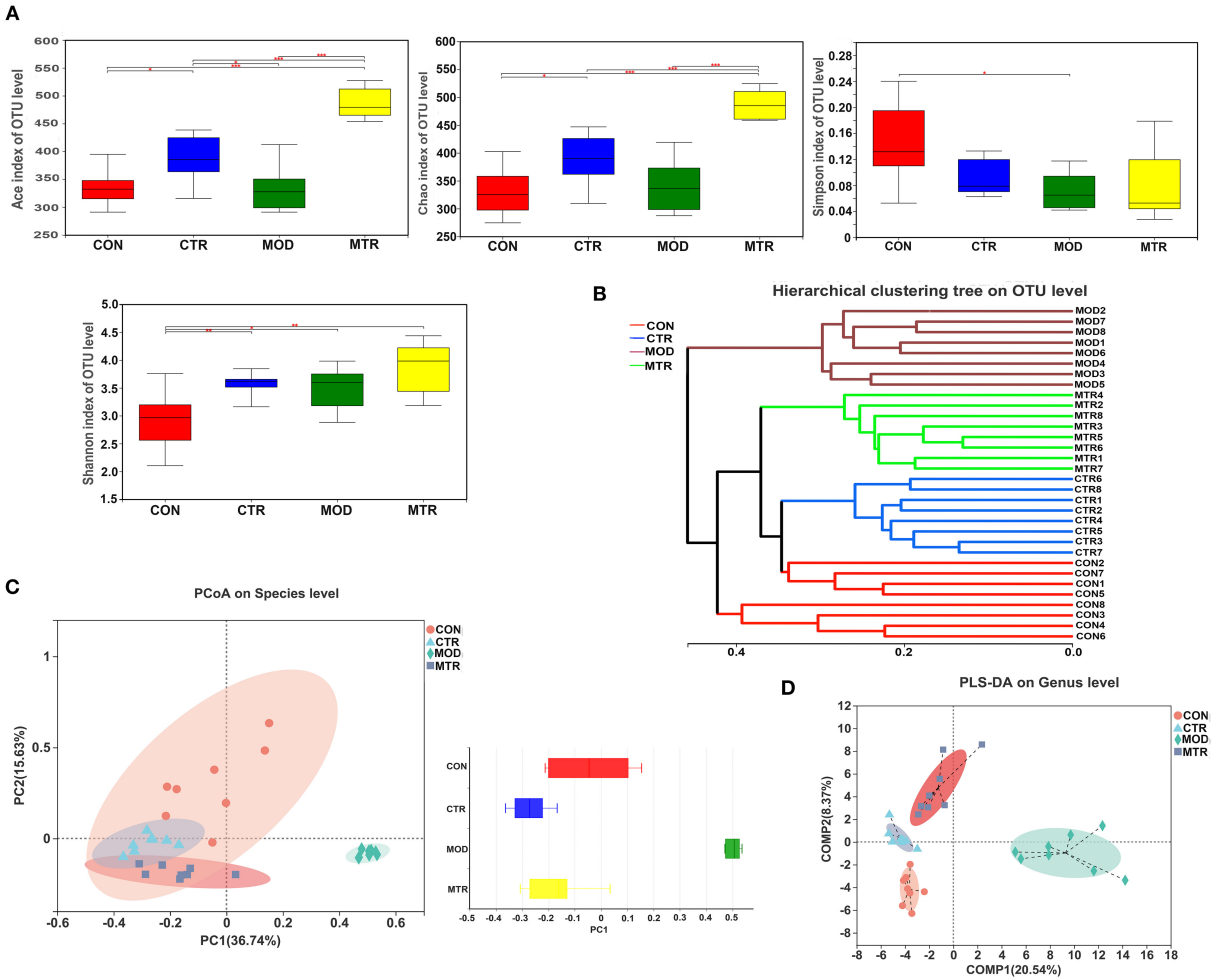
Diversity analysis of the gut microbiota among the four groups. **(A)** The alpha-diversity of the gut microbial communities. The richness and evenness of microbial community in each group were represented by the ACE, Chao, Shannon, and Simpson indices. *p* < 0.05 was considered statistically significant (*p < 0.05, **p < 0.01, and ***p < 0.005). **(B)** Hierarchical cluster tree of the microbial community in each sample. The tree was generated with Bray-Curtis distances, and each branch of the tree represents the gut bacterial community of one sample. **(C)** Principal coordinates analysis (PCoA) of the bacterial community of each sample at the species level. Principal components 1 (PC1) and 2 (PC2) explained 36.74 and 15.63% of the variance, respectively. Distances between the symbols in the plot reflect relative dissimilarities of the bacterial communities. The discrete degree of PC1 is shown in the right panel. **(D)** Partial least square discriminant analysis (PLS-DA) of the bacterial community in the four groups at the genus level. Component 1 (COMP1) and 2 (COMP2) explained 20.54 and 8.37% of the variance, respectively.

The effects of oral administration of 5-HTP on the alpha- and beta-diversity of gut microbiota in healthy mice and mice with depression-like behaviors were analyzed separately. For alpha-diversity analysis, the ACE and Chao indices that reflected the richness of bacterial species regardless of the abundance of each species, and the Shannon and Simpson indices that reflected species richness and species evenness in a microbial community were determined and compared among the four groups. As shown in Figure 2A, the ACE and Chao indices were significantly higher in the 5-HTP-treated groups (CTR and MTR) than in the untreated groups (CON and MOD), suggesting that oral administration of 5-HTP could increase the species richness of the gut microbiota in both healthy mice and mice with depression-like behaviors. Compared with the CON group, the other three groups exhibited lower Simpson indices and higher Shannon indices. These results indicated that the gut microbiota diversity of mice increased after oral administration of 5-HTP.

To assess the beta-diversity of the gut microbial communities among the four groups, a hierarchical clustering tree was generated using Bray-Curtis distance analysis. The gut microbial community of each sample was represented by one branch of the tree (Figure 2B). The samples in the MOD group clustered together and were located on different branches of the tree compared with the other three groups. PCoA analysis using the weighted UniFrac distance was performed to compare the gut bacterial communities in each sample. PC1 and PC2 accounted for 52.37% of the total variation, and the discrete degree of PC1 among the four groups is shown in Figure 2C. The MOD group showed a remarkable structural rearrangement in the gut bacterial community compared with the other three groups and was distant from the CON group, whereas the MTR group showed only slight dissimilarity with the CTR group. Both groups were located near the CON group. According to the PLS-DA analysis, compared with the CON group, significant structural differences in the gut microbial community of mice in the MOD group were detected, whereas the differences were diminished in the MTR group (Figure 2D). Taken together, our results support that depressive disorders could affect gut microbial community diversity in mice, as reported in other studies. Interestingly, our results also suggested that treatment with 5-HTP could attenuate the gut microbial community diversity changes caused by depression.

#### Composition Analysis of the Gut Microbiota in Different Groups

**Figure 3:**
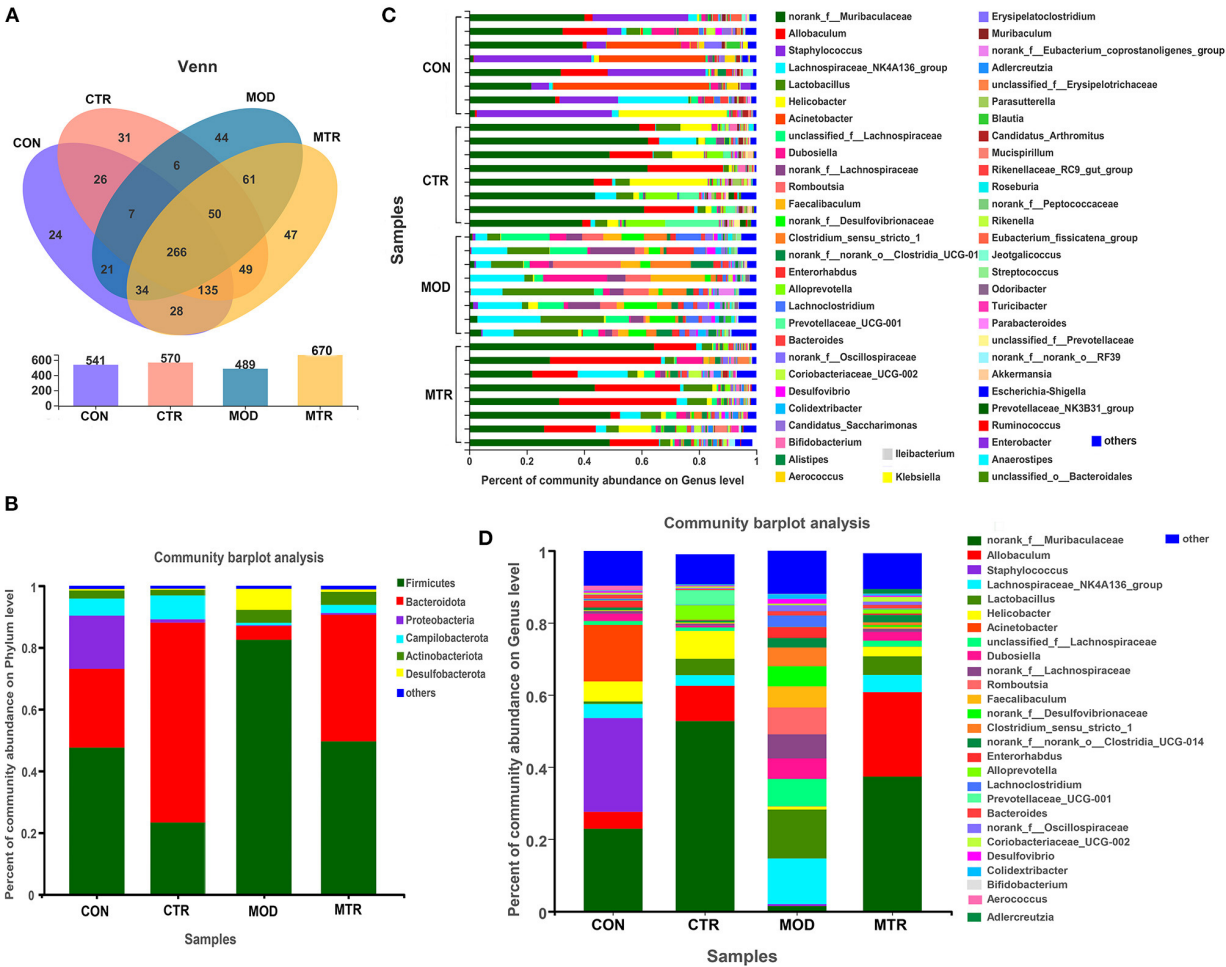
Distribution of bacterial OTUs, genera, and phyla in the four groups. **(A)** The number of operational taxonomic units (OTUs) in the four groups. Venn diagrams in the upper panel show the numbers of unique and shared OTUs among the four groups. The total number of OTUs in each group is shown in the lower panel. **(B)** Column diagrams of distribution of bacterial phyla among the four groups. **(C,D)** Column diagrams of distribution of bacterial genera in each sample and among the different groups.

The valid reads obtained in this study could be delineated into 829 OTUs with a sequence similarity cut-off threshold of 97%. The OTU numbers in the CON, CTR, MOD, and MTR groups were 541, 570, 489, and 670, respectively (Figure 3A). The number of shared OTUs among the four groups was 266, whereas the number of unique OTUs was 24, 31, 44, and 47 in the CON, CTR, MOD, and MTR groups, respectively. Based on phylogenetic information, the OTUs obtained in this study could be assigned to 14 bacterial phyla, although only six phyla had a relative abundance > 1%. The distribution of the most abundant bacterial phyla in the four groups is shown in Figure 3B. The most abundant phyla in the CON, MOD, and MTR groups were Firmicutes, and the relative abundance of this phylum in the three groups was 47.58, 82.48, and 49.57%, respectively (Supplementary Figure 2). Bacteroidetes was the most abundant phylum in the CTR group and accounted for 64.75% of the total abundance. The relative abundance of Proteobacteria in the CON, CTR, MOD, and MTR groups was 17.21, 1.07, 0.24, and 0.53%, respectively, suggesting that the relative abundance of Proteobacteria was lower in the CTR, MOD, and MTR groups than in the CON group. The relative abundance of Desulfobacterota in the MOD group was significantly higher than that in the other three groups: 0.51% (CON), 0.38% (CTR), 6.88% (MOD), and 0.80% (MTR).

The total number of bacterial genera in the gut microbial community of the four groups was 189, and the distribution of the bacterial genera in each sample is shown in Figure 3C. As shown in Figure 3D, the four groups showed significant differences at the genus level, and the structure of the gut microbial community of the MOD group was significantly reshaped compared with the other three groups. The top five abundant genera in the CON group were *Staphylococcus* (25.99%), norank_f_*Muribaculaceae* (22.88%), *Acinetobacter* (15.68%), *Helicobacter* (5.54%), and *Allobaculum* (4.70%), whereas in the CTR group they were norank_f_*Muribaculaceae* (52.75%), *Allobaculum* (9.77%), *Helicobacter* (7.72%), *Lactobacillus* (4.51%), and *Alloprevotella* (4.09%) (Supplementary Figure 3). The five most abundant genera in the MOD group were *Lactobacillus* (13.57%), *Lachnospiraceae*_NK4A136_group (12.66%), unclassified_f_*Lachnospiraceae* (7.62%), *Romboutsia* (7.45%), and unranked_f_*Lachnospiraceae* (6.69%), and the top five abundant genera in the MTR group were norank_f_*Muribaculaceae* (37.36%), *Allobaculum* (23.41%), *Lactobacillus* (5.15%), *Lachnospiraceae*_NK4A136_group (4.75%), and *Helicobacter* (2.65%). These results indicated that depression could significantly alter the abundance and composition of intestinal bacterial communities of mice at both the phylum and genus levels, and oral administration of 5-HTP could partially recover the changes in the gut microbiota of mice with depression-like behaviors.

#### Overall Changes of the Gut Microbiota in Different Groups

**Figure 4:**
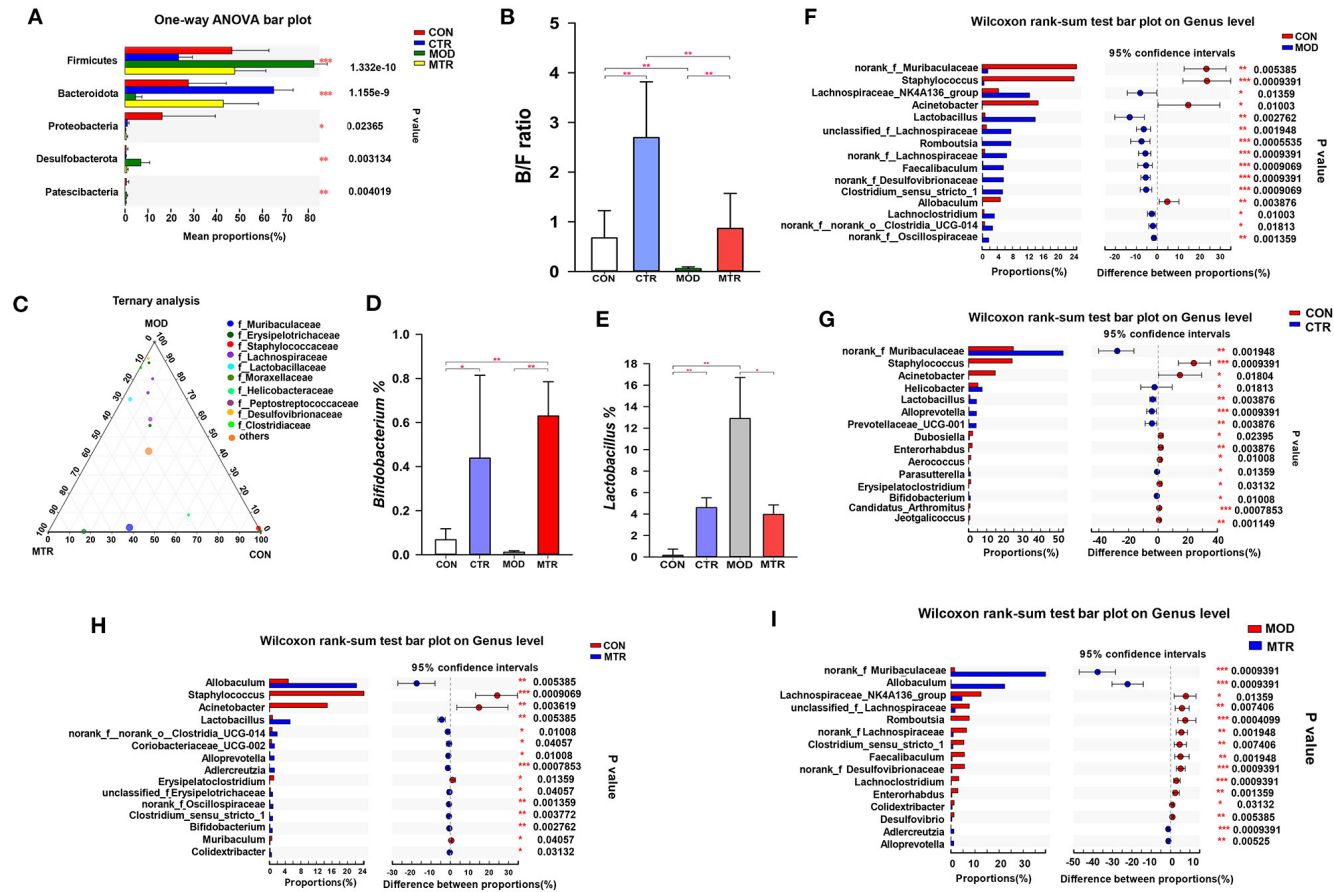
Differences in the relative abundance of specific microbial taxa among the four groups. **(A)** Significant differences in gut microbiota across the four groups at the phylum level. One-way ANOVA with a *post-hoc* Tukey-Kramer test was performed for statistical analysis. **(B)** Bacteroidetes/Firmicutes (B/F) ratios of the four groups. **(C)** The ternary plot presents the main differences in gut microbiota in the CON, MOD, and MTR groups at the genus level. Dot size indicates the relative abundance of the genera. **(D)** Relative abundance of the genera *Bifidobacterium* in the four groups. **(E)** Relative abundance of the genera *Lactobacillus* in the four groups. **(F–I)** Significant differences in gut microbiota between 5-HTP treated and untreated groups at the genus level. Wilcoxon rank-sum test was used for statistical analysis. Data are expressed as the mean and SD. *p* < 0.05 was considered statistically significant (*p < 0.05, **p < 0.01, and ***p < 0.005).

At the phylum level, Firmicutes, Bacteroidetes, Proteobacteria, Desulfobacterota, and Patescibacteria all exhibited significant variation among the four groups (Figure 4A). The relative abundance of Firmicutes was higher in the MOD group and lowered in the CTR group, whereas the relative abundance of Bacteroidetes was lower in the MOD group and higher in the CTR group. Compared with the CON group, the relative abundance of Proteobacteria was lower in the other three groups. The relative abundance of Desulfobacterota was much higher in the MOD group than in the other three groups. Compared with the CON group, the MOD group exhibited a lower Bacteroidota/Firmicutes (B/F) ratio in the gut microbiota composition, whereas both 5-HTP treated groups (CTR and MTR group) exhibited a higher B/F ratio (Figure 4B).

As shown in Figure 4C, the composition and proportional distribution of the dominant genera in the CON, MOD, and MTR groups are visually reflected in a ternary plot. The relative abundance of the genus *Bifidobacterium* was significantly higher in the CTR and MTR groups and lower in the MOD group (Figure 4D). Compared with the CON group, the relative abundance of the genus *Lactobacillus* was significantly higher in the other three groups, and the MOD group had the highest abundance (Figure 4E).

**Figure 5:**
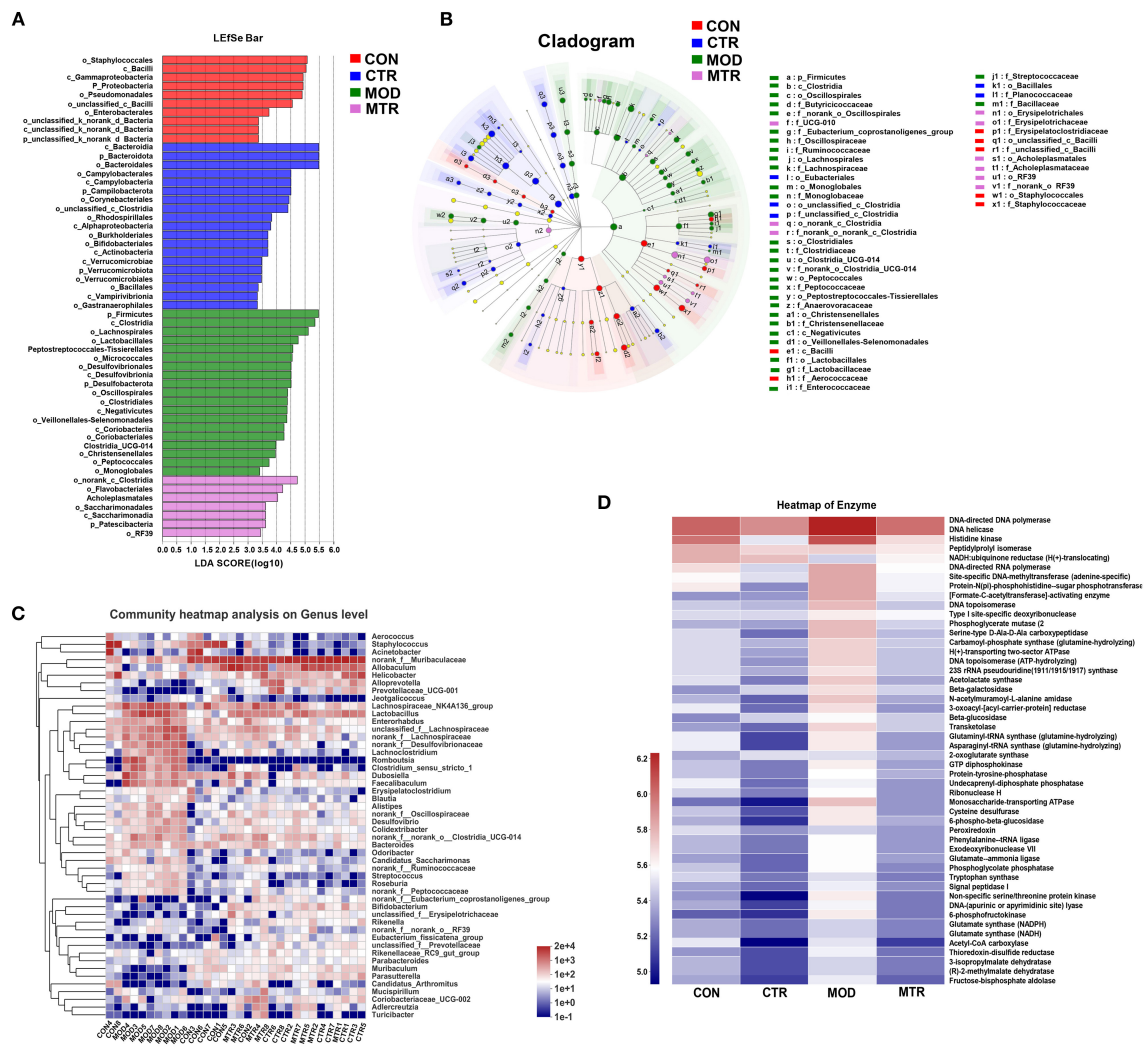
Overall changes of the gut bacteria composition and enzyme activities among the four groups. **(A)** Linear discriminant analysis (LDA) score of relative genera in the four groups. The length of the column is proportional to the taxa abundance. **(B)** LEfSe analysis of the differentially abundant taxa in the four groups. p, phylum; c, class; o, order; f, family; g, genus. **(C)** Heatmap analysis of the bacterial genera in each sample. The bacterial phylogenetic tree (shown on the left of the figure) was generated using the neighbor-joining method. The relative abundance values of bacterial genera in different samples are depicted by color intensity, and the color legend corresponding to different values is to the right of the figure. **(D)** Heatmap of different enzymes in the four groups. The relative abundance values of the enzymes in the four groups are depicted by color intensity, and the color legend corresponding to different values is to the left of the figure.

The Wilcoxon rank-sum test determined significant differences in the gut bacterial community composition between the 5-HTP treated and untreated groups at the genus level. Compared with the CON group, the abundance of four genera norank_f_*Muribaculaceae, Staphylococcus, Acinetobacter*, and *Allobaculum* were significantly lower, whereas that of 11 genera, including *Lachnospiraceae*_NK4A136_group, *Lactobacillus, Romboutsia, Faecalibaculum*, norank_f_*Desulfovibrionaceae, Clostridium*_sensu_stricto_1, *Lachoclostridium*, norank_f_norank_o_*clostridia*_UCG-014, and norank_f_*Oscillospiraceae* were significantly higher in the MOD group (Figure 4F). The CTR group exhibited a significantly higher abundance of norank_f *Muribaculaceae, Helicobacter, Lactobacillus, Alloprevotella*, and *Prevotellaceae*_UCG-001, and a lower abundance of *Staphylococcus, Acinetobacter, Dubosiella, Enterorhabdus*, and *Aerococcus* compared with those in the CON group (Figure 4G). The abundance of genera norank_f *Muribaculaceae, Allobaculum, Adlercreutzia*, and *Alloprevotella* were higher, whereas the abundance of the genera *Lachnospiraceae*_NK4A136_group, unclassified_f_*Lachnospiraceae, Romboutsia*, norank_f_*Lachnospiraceae, Clostridium*_sensu_stricto_1, *Faecalibaculum*,norank_f_*Desulfovibrionaceae,Lachoclostridium, Enterorhabdus, Colidextribacter*, and *Desulfovibrio* were significantly higher in the MTR group compared with those in the MOD group (Figure 4I). The MTR group exhibited a higher abundance of the genera *Allobaculum, Lactobacillus*, norank_f_norank_o_*clostridia*_UCG-014, *Coriobacteriaceae*_UCG-002, *Alloprevotella, Adlercreutzia*, norank_f_*Oscillospiraceae, Clostridium*_sensu_stricto_1, and *Colidextribacter*, and a lower abundance of the genera *Staphylococcus, Acinetobacter, Erysipelatoclostridium*, and *Muribaculum* compared with those of the CON group (Figure 4H). A phylogenetic tree at the genus level was generated to reflect the evolutionary relationship between the significantly altered bacteria among the four groups (Supplementary Figure 4). To distinguish the predominant taxon, LEfSe and hierarchical clustering heatmap analysis were performed to reveal the alterations in specific bacteria and the entire gut microbial community among the four groups (Figures 5A–C).

#### Prediction of Functional Changes in the Gut Microbiota of Different Groups

Based on the 16S rRNA sequencing data, the functional changes in the gut microbiota among the four groups were predicted using the COG function classification and KEGG enrichment analysis. The gut microbiota did not show a significant difference in COG function classification among the four groups but showed alterations in the abundance of microbial enzymes (Supplementary Figure 5; Figure 5D). Compared with the CON group, the MOD and CTR groups overall had a higher and lower abundance of enzymes, respectively (Figure 5D). These results could indicate that depressive disorder upregulates the metabolic activity of gut microbiota in mice, and oral administration of 5-HTP had the opposite effect.

### Effects of 5-HTP on the Serum SCFAs of Mice With Depression-Like Behaviors

**Figure 6:**
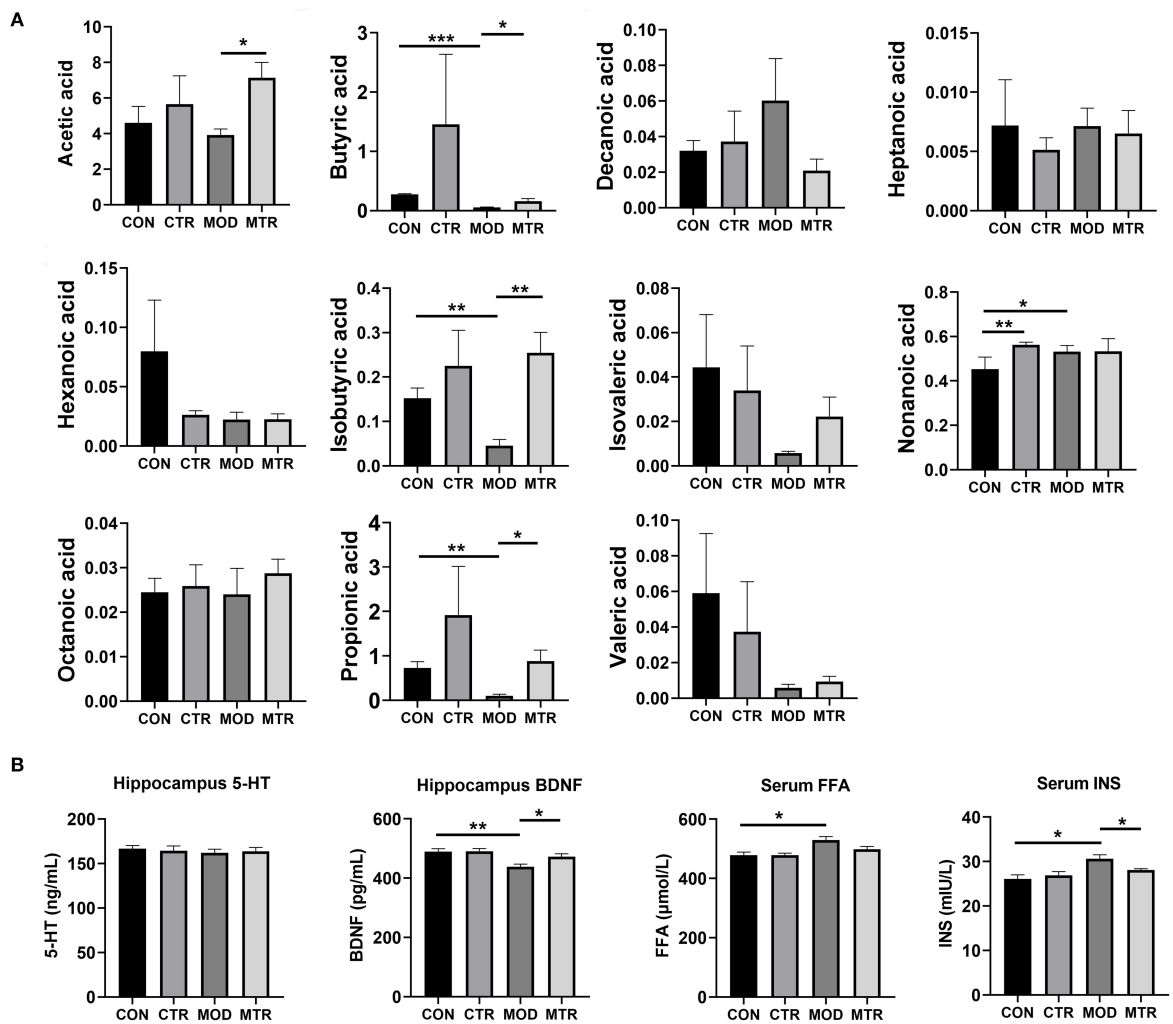
Concentrations of different SCFAs and other biochemical factors in the four groups. **(A)** The concentrations (μg mL^−1^) of acetic, butyric, decanoic, hexanoic, heptanoic, isobutyric, isovaleric, nonanoic, octanoic, propionic acid, and valeric acid in the serum samples among the four groups were determined using GC-MS. **(B)** The concentrations of 5-HT and BDNF in the hippocampus and FFA and INS in the serum samples were determined using ELISAs. Data are expressed as mean and SD. *p* < 0.05 was considered statistically significant. Data are expressed as mean with SD. *p* < 0.05 was considered statistically significant (*p < 0.05, **p < 0.01, and ***p < 0.005).

The concentrations of 11 SCFAs in the serum samples of mice in the four groups were determined using GC-MS analysis (Figure 6A). Compared with the MOD group, the amount of acetic acid in the MTR group was significantly higher. The concentration of butyric acid was significantly lower in the MOD group, and this reduction was attenuated in the MTR group compared with that in the CON group. The amount of decanoic acid in the MOD group was higher than that in the other three groups with no significance, and there were no obvious differences in the concentration of heptanoic acid among the four groups. The CON group had a higher concentration of hexanoic acid than the other three groups, but the difference was not significant. Compared with the CON group, isobutyric acid was significantly lower in the MOD group and higher in the CTR and MTR groups. The CON group had a significantly lower concentration of nonanoic acid than the other three groups. No differences were detected in the concentration of octanoic acid among the four groups. Compared with the CON group, the propionic acid concentration was significantly lower in the MOD group, and this reduction failed to occur in the MTR group. Compared with the CON group, valeric acid was lower to various degrees in the CTR, MOD, and MTR groups.

### Effects of 5-HTP on the Neurobiological Factors of Mice With Depression-Like Behaviors

The concentrations of 5-HT and BDNF in the hippocampus and FFA and INS in serum samples of mice in the four groups were measured using ELISAs. As shown in Figure 6B, there were no alterations in the level of 5-HT in the hippocampus among the four groups. Compared with the CON group, the concentration of BDNF was significantly higher in the MOD group but recovered after 5-HTP treatment in the MTR group. Serum FFA and INS levels were higher in the MOD group than in the other three groups, and the MTR group did not show significant differences compared with the CON group.

## Discussion

In past decades, researchers have investigated the impact of the gut microbiome on brain function using different strategies, including germ-free studies, antibiotic use, gastrointestinal infection studies, fecal microbiota transplantation, and probiotic treatments (Foster et al., 2017; Capuco et al., 2020). Based on these studies, mounting evidence has demonstrated that the gut microbiota could bi-directionally communicate with the brain and affect the neurological and behavioral functions through the “microbiota-gut-brain axis.” This axis connects the microbiota with the brain *via* various routes, including the immune system, gut hormone signaling, vagus nerve, tryptophan metabolism, and microbial metabolites (SCFAs) (Dinan and Cryan, 2013). Gut microbiota dysbiosis has been observed in patients with depression. Using high-throughput sequencing technology, significant alterations in the gut microbial community compositions of mice exposed to chronic stressors could be identified, and the relative abundances of *Bacteroides* and *Clostridium* were lower and higher, respectively (Bailey et al., 2011). Accumulating evidences emphasize the importance of subdiaphragmatic vagus nerve in development of depression caused by gut microbiota dysbiosis *via* the “microbiota-gut-brain axis” (Wang et al., 2020, 2021; Zhang et al., 2020; Pu et al., 2021). Our results showed that mice with depression-like behaviors had a very different community structure in their gut microbiota with the control mice, supporting these findings. Based on experimental and preclinical investigations, disturbances in the gut microbiota equilibrium have been considered involved in the pathophysiology of depression, and the regulation of the “microbiota-gut-brain axis” is becoming a new therapeutic target for the treatment of depression (Chang et al., 2022).

5-HT is a biogenic amine synthesized from 5-HTP and functions as a neurotransmitter within the brain and enteric nervous system (Gao et al., 2020). Brain 5-HT is involved in the modulation of mood and cognition, whereas 5-HT regulates gastrointestinal secretion and motility in the enteric nervous system. More than 90% of the 5-HT in the body is produced in gut mucosal enterochromaffin cells, and it cannot cross the blood-brain barrier under normal physiological conditions (Margolis et al., 2016). It is widely accepted that decreased central 5-HT availability is a major factor in developing depression (O'Mahony et al., 2015). SSRIs that block the reuptake of 5-HT from the synaptic gap are the primary antidepressant drugs in the clinic. However, because SSRIs only have limited effects on treatment-resistant depression, rebalancing the gut microbiota of patients represents a novel and attractive therapeutic method for this disease (Tian et al., 2019a). Some probiotics with the ability to regulate intestinal microecology, especially from the genera *Lactobacillus* and *Bifidobacterium*, have shown positive anxiolytic and antidepressant effects in both scientific and clinic investigations (Wang et al., 2016). The antidepressant effects of 5-HTP have been attributed to its ability to elevate the brain and the gastrointestinal tract 5-HT levels. However, the pharmacology of 5-HTP in treating other disorders, such as insomnia, chronic headaches, fibromyalgia, and obesity, remains largely unknown. Inspired by the theoretical basis of the “microbiota-gut-brain axis,” we investigated the effects of oral administration of 5-HTP on the gut microbiota of mice in this study. Our results suggested that 5-HTP could recover the disturbance in the gut microbiota equilibrium caused by depression in mice.

In the body, 5-HTP is produced from tryptophan though tryptophan hydroxylase (TPH). Tryptophan as an essential aromatic amino acid plays important roles in the “microbiota-gut-brain axis”, and its metabolism is under the direct or indirect control of the gut microbiota (Agus et al., 2018). In addition to generate 5-HT through the serotonin pathway, the vast majority of tryptophan is metabolized along the kynurenine pathway through the tryptophan-2,3-dioxygenase (TDO) or the indoleamine-2,3-dioxygenase (IDO) to produce kynurenic acid or quinolinic acid (O'Mahony et al., 2015). Kynurenic acid has neuroprotective functions, whereas quinolinic acid has neurotoxic effects. The gut microbiota could affect the kynurenine pathway indirectly by stimulating the IDO enzyme activity, or directly by synthesis of kynurenine though specific intestinal bacteria. Considered that 5-HTP could recover the disturbance in the gut microbiota of mice with depression-like behaviors, whether 5-HTP could affect “microbiota-gut-brain axis” though the kynurenine pathway needs to be further investigated.

After comparative analysis with healthy mice and mice with depression-like behaviors using PCoA and hierarchical clustering tree methods, we found that the gut microbial community diversity and species differences in the mice with depression-like behaviors were partially recovered by oral administration of 5-HTP (Figure 2). At the phylum level, after treated with 5-HTP the relative abundances of Firmicutes, Bacteroidetes, and Desulfobacterota in the mice with depression-like behaviors were comparable with those of healthy mice. However, without 5-HTP treatment, the relative abundance of these three phyla in the mice with depression-like behaviors was significantly higher or lower (Figure 4A). The B/F ratio of the gut microbial community in mice with depression-like behaviors was significantly lower than that in healthy mice, but it recovered after oral administration of 5-HTP (Figure 4B). 5-HTP also significantly increased the B/F ratio in healthy mice. A decrease in the B/F ratio has also been observed in other neuropsychiatric disorders, such as autism spectrum disorder (Strati et al., 2017). Considering that downregulation of the B/F ratio in the gut microbiome has been considered an indicator of several pathological conditions (Turnbaugh et al., 2009; Boursier and Diehl, 2015), the ability of 5-HTP to enhance the B/F ratio in the gut microbiome of mice may help improve depressive symptoms.

5-HTP also showed a strong ability to restore dysbiosis in the gut microbial community composition in mice with depression-like behaviors at the genus level. For example, compared with healthy mice, the relative abundance of norank_f_*Muribaculaceae, Lachnospiraceae*_NK4A136_group, *Allobaculum*, unclassified_f_*Lachnospiraceae, Romboutsia*,norank_f_*Lachnospiraceae, Faecalibaculum*, norank_f_*Desulfovibrionaceae, Clostridium*_sensu_stricto_1, and *Lachnoclostridium* in the gut microbial communities of mice with depression-like behaviors was significantly higher or lower (Figure 4F). Alterations in the relative abundances of these genera tended to recover in the mice with depression-like behaviors after treatment with 5-HTP (Figure 4I). Surprisingly, oral administration of 5-HTP significantly increased the relative abundance of *Bifidobacterium* in the gut microbial communities in both healthy mice and mice with depression-like behaviors (Figure 4D). Considering that some species in the genus *Bifidobacterium* have antidepressant effects, the property of 5-HTP that promotes the growth of *Bifidobacterium* in the gastrointestinal tract may contribute to its efficiency in treating depression (Wang et al., 2016).

The relative abundance of several genera in the family Lachnospiraceae, including the Lachnospiraceae_NK4A136_group, unclassified_f_Lachnospiraceae, and norank_f_Lachnospiraceae, was significantly higher in the mice with depression-like behaviors (Figure 4F). In general, Lachnospiraceae colonize the human and mouse gastrointestinal tract at a relatively high abundance and ferment plant polysaccharides into SCFAs (butyrate, acetate) and alcohols (Boutard et al., 2014; Meehan and Beiko, 2014). The spore-forming bacteria in the gut microbiota could promote host 5-HT biosynthesis by producing metabolites in the gastrointestinal tract and then regulate gastrointestinal motility and hemostasis (Yano et al., 2015; Israelyan et al., 2019). Considering that Lachnospiraceae is an obligate anaerobic spore-forming bacterium, alterations in the abundance of this family in the gastrointestinal tract could contribute to 5-HT-related-disease symptoms, including mood disorders (Yano et al., 2015).

SCFAs, as the main microbial metabolites, are key molecules in linking the host gut and brain. SCFAs play important roles in maintaining microglial homeostasis, restoring brain-blood barrier impairments, and downregulating inflammatory responses in the central nervous system (Capuco et al., 2020). SCFAs have been reported to have neuroprotective effects in mice exposed to chronic stressors by increasing the concentration of 5-HT in the brain. Our results showed significant differences in SCFA levels in mice with depression-like behaviors compared with those of the control mice (Figure 6A). The butyric, isobutyric, and propionic acid concentrations were significantly lower in mice with depression-like behaviors but partially recovered after 5-HTP treatment. Thus, 5-HTP also restored some alterations in SCFA levels caused by depression in mice. The *Allobaculum* spp. in the gut has a robust capacity to produce acetate, which is the main substrate for some SCFAs synthesis. This strain is a well-known SCFAs-producing bacteria (Tian et al., 2020; Balakrishnan et al., 2021). The relative abundance of *Allobaculum* was significantly decreased in the mice with depression-like behaviors, but restored after treatment with 5-HTP. Thus, this variation in the gut bacteria compositions might explain why the concentrations of some SCFAs decreased in mice with depression-like behaviors, but recovered after treatment with 5-HTP.

BDNF, which belongs to the neurotrophin family, is a small secreted protein that influences many processes in the brain, including the promotion of neuronal growth, formation of functional synapses, and regulation of synaptic plasticity (Foster et al., 2017). Studies have suggested that BDNF is key to learning, memory, and mood regulation and is involved in many neuropsychiatric disorders, including depression (Capuco et al., 2020). Decreased levels of BDNF have been found in the hippocampus of both humans and mice exposed to mood disorders, and treatment with several antidepressants prevented the downregulation of BDNF (Meng et al., 2020). In this study, we also observed lower levels of BDNF in mice with depression-like behaviors (Figure 6B). After the oral administration of 5-HTP, BDNF levels in the hippocampus were significantly recovered in the mice with depression-like behaviors. In addition, the increased FFA and INS levels in the serum of mice with depression-like behaviors were significantly downregulated after 5-HTP treatment. Taken together, it can be concluded that 5-HTP could restore the dysbiosis of gut microbiota in mice with depression-like behaviors and recover the dysfunction of many biochemical factors that are crucial for depression development.

Considering that depression is advancing rapidly worldwide, developing more efficient therapeutics is urgent and has great economic and social value. In recent years, a panel of probiotics has been extensively explored for their antidepressant effects in both animals and humans (Wang et al., 2016). Many studies have demonstrated that probiotics from the genera *Lactobacillus* and *Bifidobacterium* have positive effects on many psychiatric disorders, including depression, anxiety, autism spectrum disorder, and obsessive-compulsive disorder. Tian et al. (2019a) found that the species *B. longum* subsp. *infantis* E41 and *B. breve* M2CF22M7 could alleviate depression in mice in a 5-HTP synthesis and microbiota-regulating manner. Liang et al. (2015) demonstrated that treatment with *L. helveticus* NS8 could improve behavioral, cognitive, and biochemical aberrations in rats exposed to chronic restraint stress. Wang et al. (2015) claimed that *L. fermentum* NS9 could restore antibiotic-induced physiological and psychological abnormalities in rats. Tian et al. (2019b, 2020) reported that *B. breve* CCFM1025 and *B. longum* subsp. *infantis* strain CCFM687 could significantly reduce depression- and anxiety-like behaviors in mice.

The potential mechanisms by which probiotics regulate the central nervous system have been explored using different strategies, and it is now clear that probiotics could affect the central nervous system through the “microbiota-gut-brain axis” (Wang et al., 2016). Probiotics can directly alter the gut microbiota by increasing community diversity and the relative abundance of beneficial gut bacteria. Probiotics can affect the hypothalamic-pituitary-adrenal axis by altering the corticosteroid and/or adrenocorticotropic hormone levels. Probiotics can reduce pro-inflammatory cytokine production, downregulate inflammation in the immune system, affecting the endocrine and nervous systems. In addition, probiotics could also influence the levels of BDNF, γ-aminobutyric acid, 5-HT, and dopamine in the brain. However, despite these improvements in psychobiotic therapy for depression, clinical applications of probiotics in depression treatment in humans are still limited.

(R)-ketamine as a new antidepressant drug shows strong and rapid positive effects in treatment-resistant depressed patients. Based on the 16S ribosomal RNA gene sequencing, it has been revealed that (R)-ketamine could partly restore the altered compositions of the gut microbiota in a social defeat stress mouse model (Qu et al., 2017; Yang et al., 2017). In this study, we found that 5-HTP can restore gut microbiota dysbiosis and dysfunction of many biochemical factors involved in depression in mice. The results obtained in this study provide new evidence for the antidepressant effects of 5-HTP. It has been reported that administration of 5-HTP to mouse with mood disorders could restore the 5-HT levels in the enteric nervous system, and normalize the gastrointestinal motility and growth of the enteric epithelium (Israelyan et al., 2019). The accurate mechanisms that 5-HTP regulates the gut microbiota dysbiosis in mice with depression-like behaviors are not elucidated in this study, and awaits future experimental endeavor. Considering that 5-HTP in a slow-release formulation has a synergistic effect with the SSRIs in the treatment of depression, we believe that 5-HTP has great potential in developing next-generation antidepression drugs (Jacobsen J. P. R. et al., 2016).

## Data Availability Statement

The datasets presented in this study can be found in online repositories. The names of the repository/repositories and accession number(s) can be found below: https://www.ncbi.nlm.nih.gov/, PRJNA782400.

## Ethics Statement

The animal study was reviewed and approved by Southwest Medical University Ethics Committee.

## Author Contributions

Experiments were designed and supervised by XG and performed by LW, LR, YW, ML, JZ, FK, FW, JY, and XL. Data were analyzed by XG. The manuscript was initially drafted by XG and revised by LL and QW. All authors have read and approved the final manuscript.

## Funding

This work was supported by the National Natural Science Foundation of China (Grant No. 32160579), the China Postdoctoral Science Foundation (Grant No. 2020M683364), the Department of Science and Technology of Sichuan Province (Grant No. 2020YJ0129), the Jiangxi Natural Science Foundation (Grant No. 20202ACBL215001), and the Collaborative Fund of Science and Technology Agency of Luzhou Government and Southwest Medical University (Grant No. 2020LZXNYDJ29, 2021ZKMS045).

## Conflict of Interest

The authors declare that the research was conducted in the absence of any commercial or financial relationships that could be construed as a potential conflict of interest.

## Publisher's Note

All claims expressed in this article are solely those of the authors and do not necessarily represent those of their affiliated organizations, or those of the publisher, the editors and the reviewers. Any product that may be evaluated in this article, or claim that may be made by its manufacturer, is not guaranteed or endorsed by the publisher.
