# Supplementary material for: Oral Administration of 5-Hydroxytryptophan Restores Gut Microbiota Dysbiosis in a Mouse Model of Depression
Source: Front Microbiol. 2022 Apr 28;13:864571. doi: 10.3389/fmicb.2022.864571 (PMC9096562; doi:10.3389/fmicb.2022.864571)
Supplement: Supplementary file 1 [file Data_Sheet_1.docx]

1. **Methods and materials**

**1.1 Chronic unpredictable mild stress (CUMS)**

The CUMS has been widely used to induce depression-like behaviors in mouse. The detailed protocols for CUMS used in this study are as follows:

[1] Shaking: Mice are placed in the cage and shaken horizontally for 5 minutes.

[2] Tilt: Place one side of the cage on a tilting rack at an angle of 45° for 12 hours.

[3] Noise: 80db noise for 30 minutes.

[4] Swimming: Place the mice in a bucket filled with water at 25±1°C (depth 8 cm) so that the mice's hind toes can just touch the bottom of the bucket. after 5 minutes, remove the animals and place them back in the cage.

[5] Restraint: The mice are placed in a home-made restraint tube (modified from a 50 ml centrifuge tube with sufficient ventilation holes drilled around the tube and a cap drilled to hold the mice's tails in place) for 12 hours.

[6] Fasting for 12 hours with access to water.

[7] Wet cage: place the mice in a nest with a humidity of approximately 70% for 24 hours.

[8] Odour: Place coriander, garlic, onion and other odorous items in the cage and stimulate for 24 hours.

[9] Empty cage: Remove the bedding from the rat cage for 24 hours.

[10] Day-night reversal: Place the animals in the day-night reversal box at 8:00 am and keep them in the dark without lights; at 20:00 pm, turn on the fluorescent lights in the day-night reversal box so that the animals are in the light state until they are removed at 8:00 am the next day.

Two CUMS procedures were used per day for the first four weeks, and one CUMS procedure for another week without repetition for two consecutive days.

**1.2 Behavioral tests**

[1] Forced swimming test: the mouse was put into a round beaker with 23-25℃ warm water. The diameter of the beaker is 10 cm and the depth of the water is 10 cm, which could make sure that the mice's hind feet cannot touch the bottom of the bucket. After 5 min the mice are taken out of the beaker and put back into the cage.

[2] Sucrose preference test: The mice were habituated to sucrose and water bottles randomly for 72 h before testing. After fasted for 24 h, the mouse were separated in a single per cage equipped with two drinking bottles: one containing 1% sucrose and the other water. Sucrose preference = [Sucrose solution intake/(Sucrose solution+Water) intake]×100%.

1. **Results**

**Table S1. Information of valid reads of 32 samples in this study.**

| Sample | Sequence number | Base number | Mean length | Minimal length | Maximal length | Coverage (%) |
| --- | --- | --- | --- | --- | --- | --- |
| CON1 | 32187 | 13596754 | 422 | 277 | 433 | 99.73 |
| CON2 | 30647 | 12890132 | 420 | 258 | 432 | 99.70 |
| CON3 | 31583 | 13417981 | 424 | 278 | 487 | 99.76 |
| CON4 | 45815 | 19525325 | 426 | 283 | 432 | 99.81 |
| CON5 | 35344 | 14996965 | 424 | 283 | 432 | 99.80 |
| CON6 | 30971 | 13218219 | 426 | 342 | 432 | 99.73 |
| CON7 | 32165 | 13449588 | 418 | 259 | 462 | 99.68 |
| CON8 | 32356 | 13499065 | 417 | 336 | 445 | 99.77 |
| CTR1 | 35170 | 14817548 | 421 | 232 | 482 | 99.77 |
| CTR2 | 30884 | 12943922 | 419 | 264 | 452 | 99.68 |
| CTR3 | 31009 | 13079291 | 421 | 221 | 432 | 99.79 |
| CTR4 | 33358 | 14137029 | 423 | 207 | 441 | 99.82 |
| CTR5 | 32021 | 13400412 | 418 | 274 | 438 | 99.68 |
| CTR6 | 32116 | 13490557 | 420 | 299 | 436 | 99.59 |
| CTR7 | 32970 | 13927769 | 422 | 210 | 450 | 99.66 |
| CTR8 | 32438 | 13695914 | 422 | 230 | 455 | 99.77 |
| MOD1 | 30898 | 12745965 | 412 | 266 | 454 | 99.73 |
| MOD2 | 33139 | 13635225 | 411 | 362 | 457 | 99.78 |
| MOD3 | 30903 | 12755539 | 412 | 278 | 485 | 99.81 |
| MOD4 | 41001 | 17078276 | 416 | 259 | 469 | 99.71 |
| MOD5 | 31177 | 12935431 | 414 | 219 | 432 | 99.66 |
| MOD6 | 37886 | 15568692 | 410 | 297 | 432 | 99.77 |
| MOD7 | 32900 | 13634189 | 414 | 214 | 431 | 99.79 |
| MOD8 | 35083 | 14591076 | 415 | 231 | 509 | 99.84 |
| MTR1 | 31823 | 13442720 | 422 | 257 | 460 | 99.70 |
| MTR2 | 32340 | 13643761 | 421 | 238 | 503 | 99.79 |
| MTR3 | 44719 | 18932437 | 423 | 258 | 488 | 99.75 |
| MTR4 | 38083 | 15836128 | 415 | 258 | 488 | 99.68 |
| MTR5 | 35300 | 14939994 | 423 | 259 | 446 | 99.76 |
| MTR6 | 36841 | 15564779 | 422 | 258 | 509 | 99.88 |
| MTR7 | 33838 | 14167674 | 418 | 232 | 432 | 99.74 |
| MTR8 | 39672 | 16611801 | 418 | 314 | 467 | 99.84 |


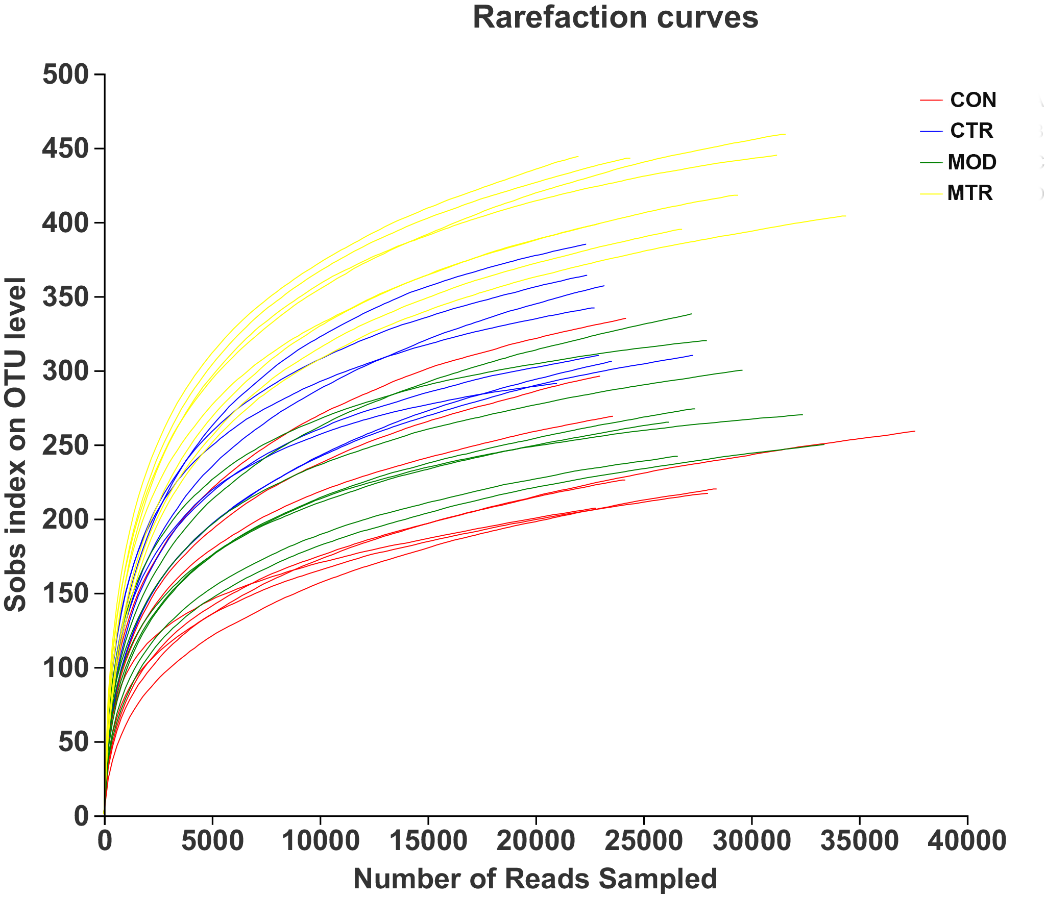


**Figure S1.** **Rarefaction analysis of different samples.** Rarefaction curves of the different samples performed high-throughput sequencing. OTUs are identified using 97% cutoffs. Total sample richness is presented as the richness estimator Sobs index. The curve was generated by plotting the number of valid sequences for each sample against the Sobs index of OTUs.


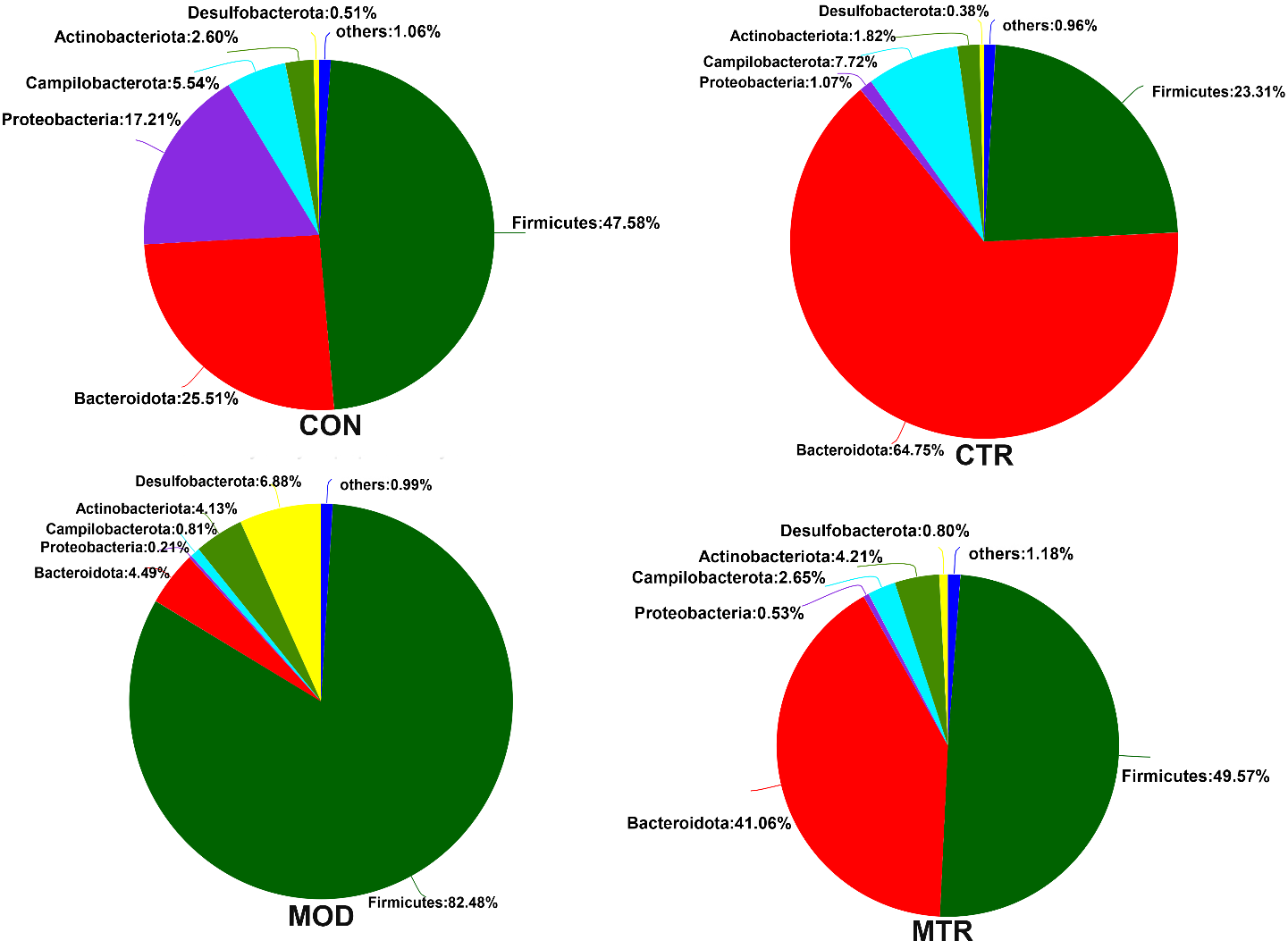


**Figure S2. Distribution of bacterial phyla among the four groups.** The pie diagrams show the bacterial composition of the four groups, and the relative abundances of the bacterial phylum in each group are shown. Others represent bacteria with less than 0.1% abundance at the phylum level.


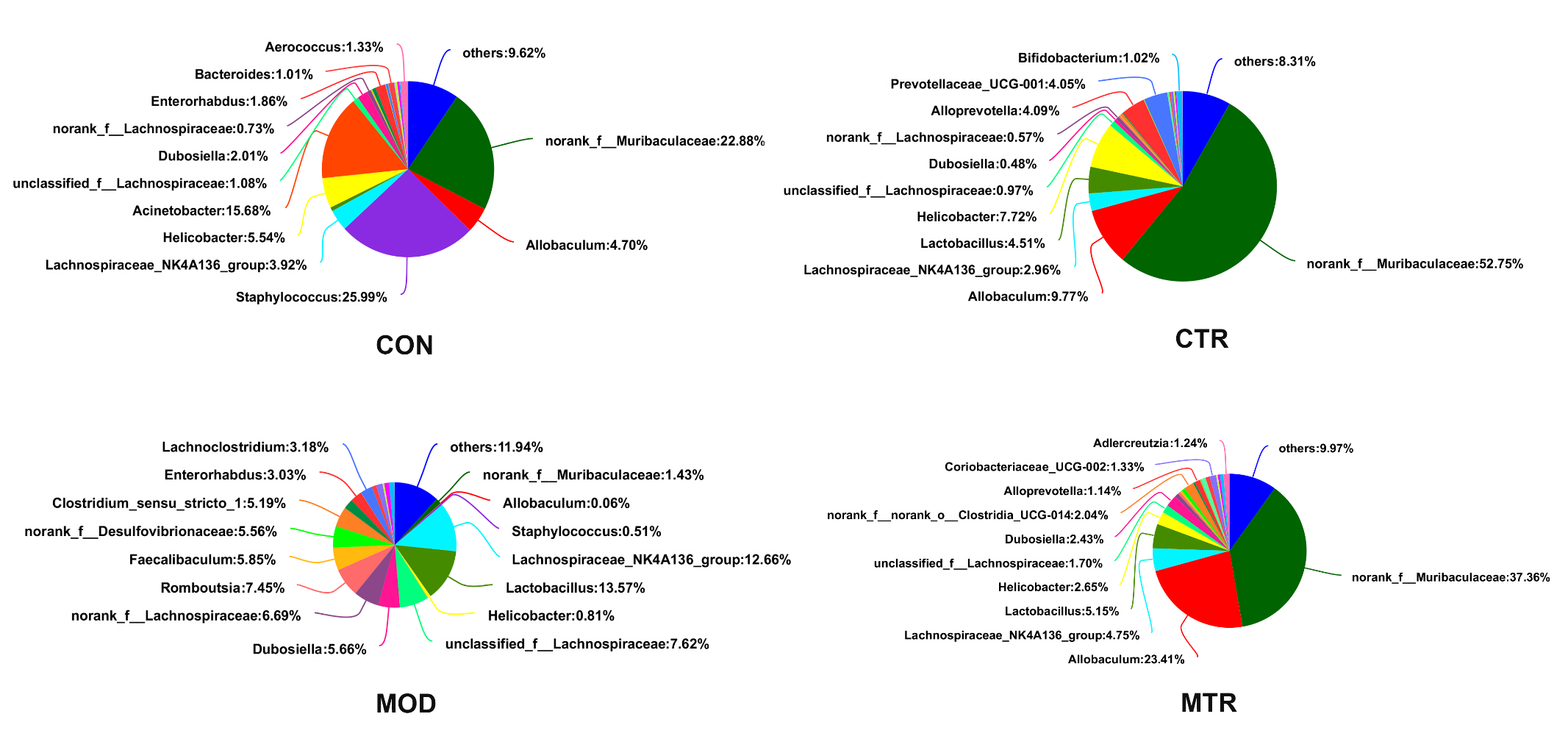


**Figure S3. Distribution of bacterial genera among the four groups.** The pie diagrams show the bacterial composition of the four groups, and the relative abundances of the bacterial genus in each group are shown. Others represent bacteria with less than 0.1% abundance at the genus level.

**
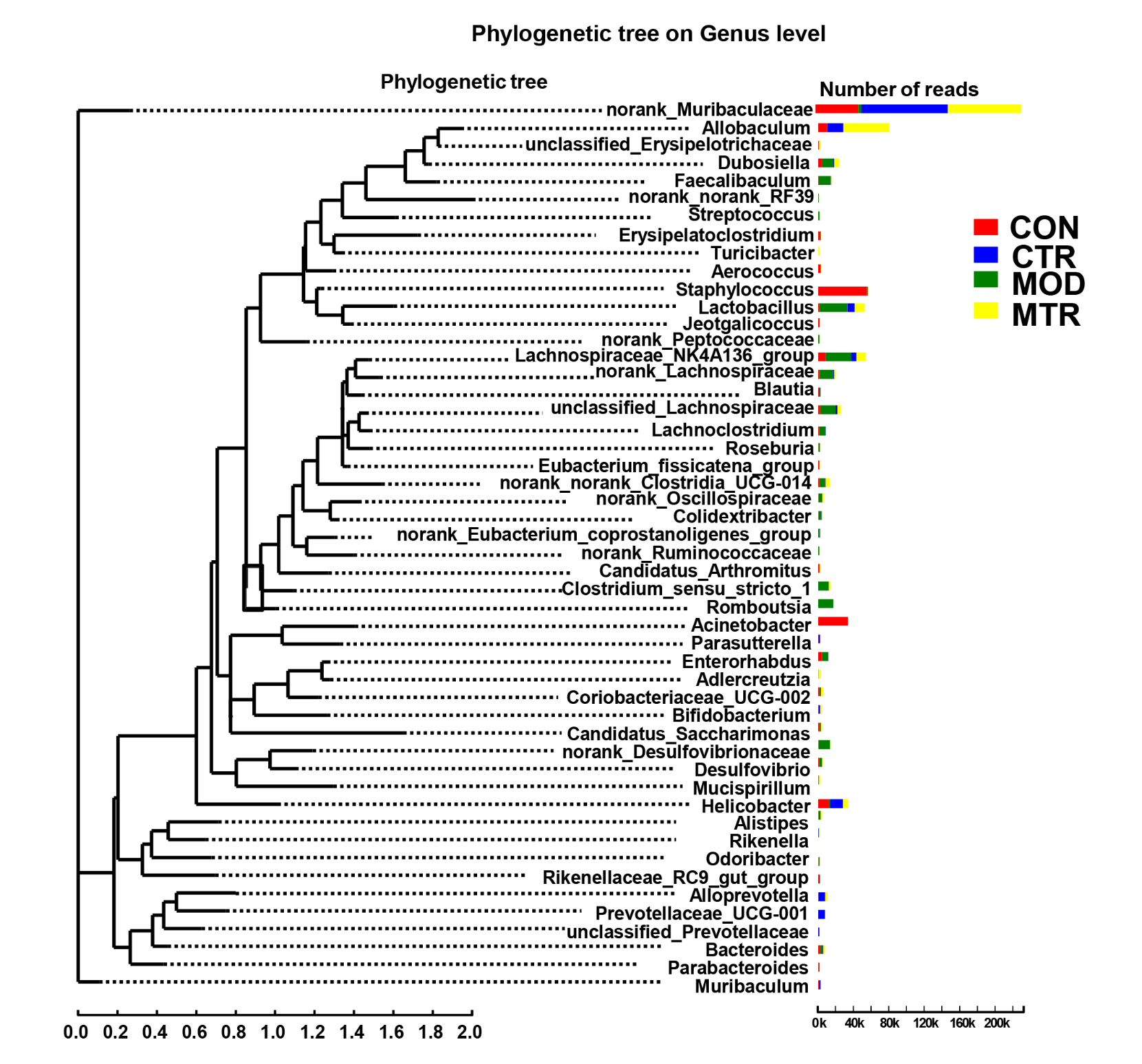
Figure S4 Phylogenetic tree of bacterial genera in the four groups.** The number of reads of bactera genera in the four groups were shown in the righr panel.


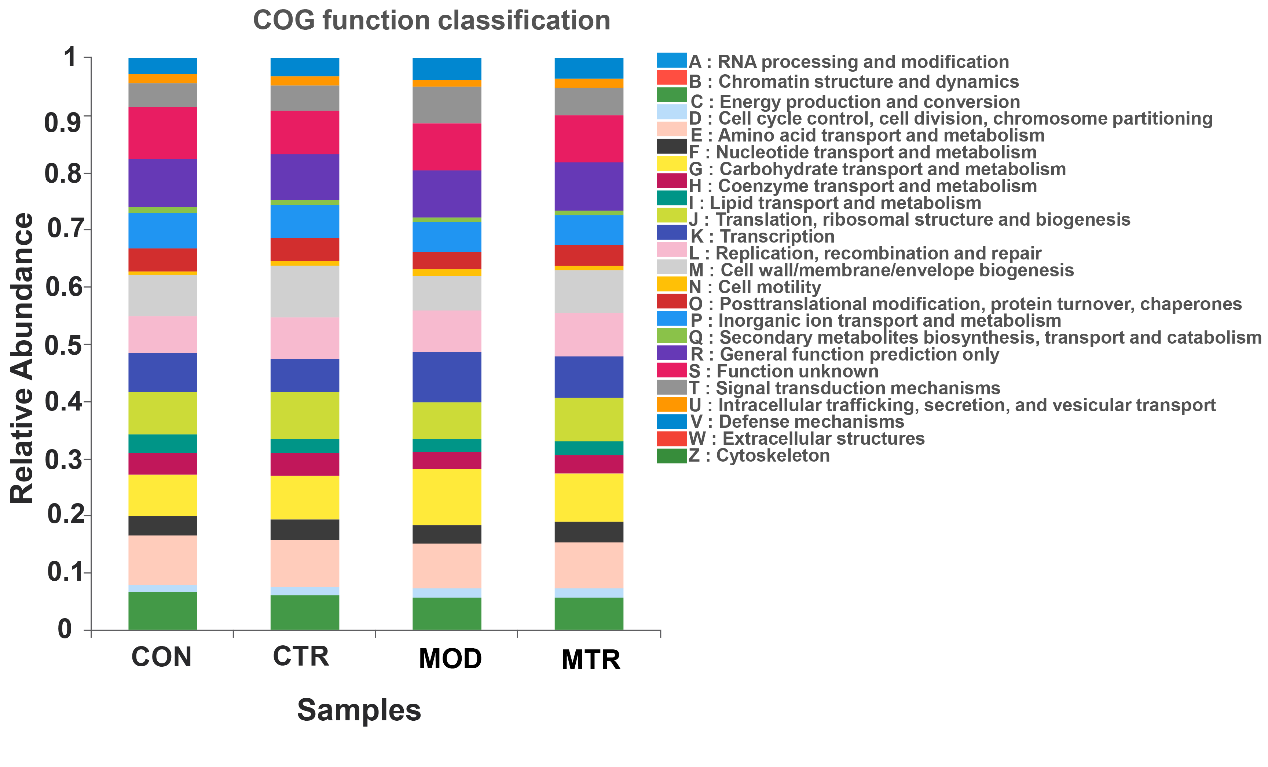


**Figure S5. Predicted functional changes of the gut microbiota in the four groups.** COG classification analysis of the gut bacterial community in the four groups. Different colors present the relative abundances of different functions.
